# Understanding #WorldEnvironmentDay User Opinions in Twitter: A Topic-Based Sentiment Analysis Approach

**DOI:** 10.3390/ijerph15112537

**Published:** 2018-11-13

**Authors:** Ana Reyes-Menendez, José Ramón Saura, Cesar Alvarez-Alonso

**Affiliations:** 1 Department of Business Economics, Faculty of Social Sciences and Law, Rey Juan Carlos University, Paseo Artilleros s/n, 28032 Madrid, Spain; ana.reyes@urjc.es; 2 Institute for Global Law and Policy, Harvard Law School, Harvard University, Cambridge, MA 02138, USA; calvarezalonso@law.harvard.edu

**Keywords:** #WorldEnviromentDay, sentiment analysis, Twitter, Python, Sustainable Development Goals, machine learning, Nvivo, textual analysis

## Abstract

The main objective of this exploratory study is to identify the social, economic, environmental and cultural factors related to the sustainable care of both environment and public health that most concern Twitter users. With 336 million active users as of 2018, Twitter is a social network that is increasingly used in research to get information and to understand public opinion as exemplified by Twitter users. In order to identify the factors related to the sustainable care of environment and public health, we have downloaded *n* = 5873 tweets that used the hashtag #WorldEnvironmentDay on the respective day. As the next step, sentiment analysis with an algorithm developed in Python and trained with data mining was applied to the sample of tweets to group them according to the expressed feelings. Thereafter, a textual analysis was used to group the tweets according to the Sustainable Development Goals (SDGs), identifying the key factors about environment and public health that most concern Twitter users. To this end, we used the qualitative analysis software NVivo Pro 12. The results of the analysis enabled us to establish the key factors that most concern users about the environment and public health such as climate change, global warming, extreme weather, water pollution, deforestation, climate risks, acid rain or massive industrialization. The conclusions of the present study can be useful to companies and institutions that have initiatives related to the environment and they also facilitate decision-making regarding the environment in non-profit organizations. Our findings will also serve the United Nations that will thoroughly review the 17 SDGs at the High-level Political Forum in 2019.

## 1. Introduction

New technologies allow users to express their opinions in the so-called social media, i.e., online platforms where users create communities around a topic of interest [1]. Owing to these new technologies, it is now possible to obtain information, carry out research on the expressed sentiments, or extract the key factors, the specific factors that receive special interest from a specific community, that serve both the users and the institutions that develop the agenda of actions vis-à-vis these problems [2,3].

One of such topics is the environment, this particular concern for the environment and how it can affect people’s health is well grounded [4,5]. Ever since pioneers of sustainable development began to study the impact of human activities on the environment [6,7] several decades ago, the global situation has progressively worsened. By now, it has reached the point where natural resources are being consumed at the rate which is 40% higher than what the Earth can endure according to [8]. The increase in this kind of events, is linked to climate change and the environment in general, so that research like this, can help eradicate this type of events by providing information and analysis methodologies for future research or studies in this field.

Furthermore, nowadays, it is essential to not only manage the resources but also to ensure that these resources will be available for future generations and to minimize the impact of human activities on the environment and public health [7,8,9].

Taking into consideration that sustainable management of resources is of paramount importance [10], institutions and their leaders are creating principles named Sustainable Development Goals (SDGs), to ensure the viability of the planet.

### 1.1. Sustainable Development Goals

In a meeting convened by the United Nations Organization (UN) that took place in 1992, world leaders established the principles of the Rio Declaration and the Agenda 21, a document of principles which laid the foundations for the sustainable development of the 21st century. The meeting was attended by more than 150 heads of State. It was in 2000 when the United Nations Educational, Scientific and Cultural Organization (UNESCO) created the Millenium Development Goals (MDGs).

In this new approach, the agendas of the United Nations Development Programs (UNDP) have been collected, along with those of the United Nations Environment Program (UNEP), World Health Organization (WHO), United Nations Children’s Fund (UNICEF), UNESCO, as well as other development agencies [11].

Since then, the interest in the environment and public health have become the main concerns for society [6]. From that moment, people started to be considered as a fundamental part of sustainable development and, consequently, governments and institutions started to invest in them, in their health, and in the environment that supports their existence [7], as summarized in the Agenda 21 promulgation: “Human beings are the center of concerns for sustainable development; they have the right to a healthy and productive life in harmony with nature” [12].

After the meeting, numerous international institutions have sought to give continuity to the principles that bring together the environment, health, and sustainable development, such as the one organized by the World Health Organization [13].

All these developments at the national and international political levels have promoted a holistic perspective on health, economy, and environment. Along with economy [14] and decision-making [15], health is now becoming a global concern for almost all sectors of the society.

With the global aim of promoting sustainability, public health, and the environment, in 2015, the United Nations presented the SDGs, where 17 principles of sustainable development based on Agenda 21 were formulated emphasizing equality, human rights, and non-discrimination of people [3,16,17,18]. Some of the principles include Climate Action, Life on Land, or Clean Water and Sanitation, were formulated [19,20,21] (see Table 1 for the complete list). Yet, to what extent does the society share the concerns about environment and public health of the SDG leaders?

Simultaneously with the development and implementation of the principles of sustainable development, the use of new Information Communication Technologies, such as social media and mobile-based communications, is revolutionizing the way users and businesses communicate with each other [22,23]. Here, the use of inadequate communication strategies is responsible for the low impact that efforts related to the environment have had [24]. In this sense, the adequate use of technology can facilitate the communication tasks about the environment, and, by doing so, help to achieve sustainable [1] and effective management [25]. In addition, the use of new information and communication technologies makes it possible to get a comprehensive picture of user concerns and to identify the most important factors involved therein [26,27].

Consequently, the main objective of this exploratory study is to identify the social, economic, environmental and cultural factors related to the sustainable care of both environment and public health that most concern Twitter users. The results of the analysis enabled us to establish the key factors that most concern users about the environment and public health.

### 1.2. Twitter as a Social Communication Platform to Measure Public Opinion

In recent years, social media has emerged as a virtual space where users can express their concerns about both the environment and health and other issues of public interest [28,29,30,31]. Actually, the use of social media to develop daily activities is very high worldwide, being an essential source of information on the communications and opinions of users on the Internet [24]. Every minute, some 473,000 tweets are published, 3,788,140 searches are performed on Google, and 18,055,555 text messages are sent [24].

Among social media, the one that most favors users’ sharing their opinions is Twitter [25]. With its 336 million active users, Twitter is increasingly useful for the extraction of factors from public opinion in social media [28,32]. This social network serves as an optimal starting point, since, unlike Facebook that offers limitations with private or semi-private profiles, Twitter facilitates open communication between users and allows them to share their opinions in a maximally accessible way publicly, main advantage with respect to other social networks that are private [33].

Twitter allows users to create and share opinions on the spur of the moment. It also allows users to interact with each other either by following other users, or by visiting their profiles and sharing the same hashtag about a common topic of interest. A hashtag is a tag that is represented by a pad (#) and that allows users to group all the published tweets on a given topic [34]. The comments with a hashtag can be visualized together; their retweets can be visualized as well. A retweet is a direct sharing of a comment written by other users, as what they write is aligned with the opinion of the person who does the retweet so that the comment appears directly in his/her profile. The most used hashtags become topics of general interest, or trending topics [35].

Table 2 was created by the authors based on Thapa [36] and Pena [37] to show the most used hashtags when earthquakes were produced in different parts of the world. In the case of the Mexican earthquake, the hashtags #sismo and #FuerzaMexico were the most frequently used, highlighting thus the trending topics in that country throughout the year 2017. Regarding the earthquake that hit Haiti in 2010 and shocked the world, #Haiti was the most shared hashtag throughout the world during that year. Furthermore, with respect to the earthquake in Nepal, during the month following the catastrophe, 33,610 tweets were published with the hashtag #nepalearthquake [36]. These figures show how the comments are grouped with respect to a topic around a series of hashtags shared by other users and can be studied to obtain information [30].

Among the first to study Twitter as a new means of communication were Java et al. [31] who highlighted that this social network is a space to share and search for information, as well as a space where users with similar interests can meet and form communities. Thereafter, Twitter has become increasingly used for research purposes [25], including the studies on public opinion [30]. In addition, Twitter can be used by companies to identify the key factors about environment and public health that can help them improve their management and provide user insights as it is explained in the discussion section of this study [32,33]. Unlike other social media such as Facebook, the growth statistics of Twitter are positive, the number of its active users is growing every month based on Aggarwal [34]. Therefore, Twitter is a very relevant social network [38].

## 2. Literature Review

Since the formulation of the SDGs as reference points for sustainable development, several studies took them as a reference [9,39]. This is due to the need of quantitative measures that the governments can rely on to assess their performance with respect to the challenges posed that range from natural resources management to pollution control. 

When researchers aim to get information for governmental decision-making about a certain topic, one the means they use is to assess the opinion of individuals or users in social media [3,32].

Even there is a growing community in social media like Facebook or Twitter and each time more individuals have a voice on these networks about such topics as the environment, this media has shown its weaknesses as a single means for policy-making as not all the individuals are represented by social media users [40].

However, there are numerous studies have used Twitter and hashtags to identify topic-based feelings of the population regarding a specific issue related to the environment and public health [32,38]; other studies, e.g., those investigating public opinion in social media after a natural disaster, analyzed the keywords extracted from the comments [41]. Table 3 gives an overview of the object of our research based on previous important related work.

Among other relevant studies is the research by Woo et al. [41] that examined how Twitter users used hashtags to communicate during an earthquake or a flood since they could not make phone calls. Also, Chisholm and O’Sullivan [44] analyzed the hashtag #characterbuildings used to express Twitter users’ views on sustainable housing in New Zealand. Likewise, Saura et al. [2] used published tweets to identify social, economic, environmental and cultural factors that allow hotel establishments to improve decision-making and management of sustainability.

Tlebere et al. [44] developed a model of social awareness about the environment based on social media with the aim of providing people with the knowledge, skills, and attitudes necessary to reduce adverse environmental impact. Furthermore, Ekenga et al. [28] used the social platform to measure public sentiment about environmental health, obtain information on the issues that most concern the community, and communicate with them about environmental risks.

In the present study, following Chisholm and O’Sullivan [32] and Palomino et al. [38], we will focus on public opinion about the environment through the analysis of the hashtag #WorldEnvironmentDay in Twitter. #WorldEnvironmentDay was the most used hashtag to express opinions about the Environment on the day of the environment so that it joins the opinions of Twitter users about the topic. Methodologically, we will follow Saura et al. [2] by using sentiment and textual analyses. Firstly, we have downloaded tweets that used the hashtag #WorldEnvironmentDay on the respective day. As the next step, sentiment analysis was applied to the sample of tweets to group them according to the expressed feelings. Thereafter, a textual analysis was used to group the tweets according to the SDGs, identifying the key factors about environment and public health that most concern Twitter users. To this end, we used the qualitative analysis software NVivo Pro 12 (QSR International, Melbourne, VIC, Australia). The results will help identify the key factors about environment and public health that most concern the public (Twitter users). Thus, the main objective of this exploratory study is to identify the social, economic, environmental and cultural factors related to the sustainable care of both environment and public health that most concern Twitter users. Therefore, we have structured this study in the following parts: Introduction, Materials and Methods, Methodology, Results, Discussion and Conclusions.

### 2.1. Sentiment Analysis Approaches for Social Network Analysis

Sentiment analysis is an increasingly used methodology to identify the emotional burden contained in the messages published on a given topic [28,45]. Deriving from the use of Big Data, it is used to conduct research on the impact of events on social media, to measure the opinion of products and services, as well as to understand communication in online environments [38,46]. In essence, sentiment analysis involves downloading topic-based comments published on Twitter. After that, we can analyze user opinions of the users expressed in these comments using the models developed with machine learning [47].

Sentiment analysis can be combined with other technologies to extract the most important factors under study [48]. For instance, Pak and Paroubek [49] developed several different methodologies that can be used in Twitter analysis. In general, the analysis takes into account the connotations of the frequently recurring words, which signal different feelings and can be classified into positive, negative, or neutral. For example, Palomino et al. [38] used sentiment analysis to identify public opinion and study the related discourse to understand the impact of the natural environment on people’s health and well-being. On the other hand, Ekenga et al. [28] conducted a topic-based sentiment analysis of Twitter comments and demonstrated that this social media platform serves to measure public sentiment about environmental health, to obtain information on the issues that most disturb the community, and to communicate about environmental risks. All in all, previous research has demonstrated that social media are a good platform to perform this type of analysis [28,49]. Although some of the challenges of sentiment analysis are those related to the context in which content is analyzed, sarcasm or ironies, this technology can be trained to mitigate these effects and diminish these weaknesses. Precisely, with the use of machine learning and data mining processes we can anticipate ironies and sarcasms to anticipate this problem. In this research, those tweets that have been classified as sarcasm, ironies or in unknown contexts have been classified as neutral. This fact, is still a challenge and a limitation to improve the development of new technologies in research and not to validate this type of process that allows much more advanced analysis and with higher volumes of data than can be analyzed by the human being [3].

### 2.2. Textual Analysis

Textual analysis is a type of qualitative analysis that is applied to a text with the aim of grouping the concepts under study into “nodes”. This approach can be applied to an event, a company, or any other object of study [50]. The software most used to perform this analysis is NVivo (QSR International, Melbourne, Australia) [2,51]. The outcome of the method is grouping of the concepts in nodes that are very useful to perform exploratory analysis [52]. This approach has a greater descriptive capacity than if the software had not been used [53,54].

The nodes are distributed in different hierarchical levels. At the first level, there are concepts that are conceptually independent of each other. At the second level, there are the branches that start with each of the nodes and follow a hierarchical organization. Finally, there are the indicators that can be extracted from the research results and that are related to the aim of the specific study [52]. One of the relevant studies where textual analysis was used to identify factors related to the environment was conducted by Saura et al. [2]. Each of the three nodes identified by the authors corresponds to negative factors, positive factors, and neutral factors.

## 3. Conceptual Framework and Hypothesis Development

As already highlighted in the previous sections, social media have been increasingly used to obtain information about public opinion, sentiment, and key factors related to various objects of study. For instance, Chisholm and O’Sullivan [32] used hashtags to identify Twitter user sentiment. Similarly, Palomino et al. [38] used the social network to identify public opinion through the analysis of the tweets of a hashtag and to study the feelings expressed in the discourse related to nature-deficit disorder and other nature-heath concepts. In this sense, to validate that it is possible to identify sentiments on Twitter related to a specific topic and based on the previous research, the following hypothesis can be formulated:

**Hypothesis 1** **(H1).** *The hashtag #WorldEnvironmentDay serves to identify the feelings of users about the environment.*

Furthermore, Woo et al. [41] performed the analysis of public opinion through the study of the tweets published in relation to a natural disaster in Korea. Based on the relevant comments, the authors identified how Twitter users’ sentiment changed and how topic-based keywords related to the natural disaster appeared. Likewise, in their analysis of comments on Twitter profiles of hotels, Saura et al. [2] identified the most important factors related to the environment. Consequently, with the objective of identifying important factors for the environment, we propose the following hypothesis based on the similar above-mentioned research:

**Hypothesis 2** **(H2).** *The analysis of the communications of users who use the hashtag #WorldEnvironmentDay can be used to establish the key factors related to the environment.*

Next, the starting point for the studies by Messerlin et al. [26] and Browman and Stergiou [9] was, among other topics of public interest, the SDGs with their 17 principles related to the environment to identify the key factors about environment and public health. On the other hand, Kumar et al. [3] selected the SDG related to health and the environment for their analysis. Therefore, with the aim of being able to link the important factors related to the SDG established by the United Nations, we propose the following hypothesis:

**Hypothesis 3** **(H3).** *The communications of users on Twitter through the hashtag #WordEnvironmentDay during the respective day are related to Sustainable Development Goals (SDGs).*

## 4. Methodology

The first of the two methodologies employed in the present study is sentiment analysis of the tweets published by users on Twitter with the hashtag #WorldEnvironmentDay. Following Palomino et al. [38] and Saura et al. [2], firstly, we developed a sentiment analysis. The second methodology was textual analysis, in which the qualitative analysis software NVivo Pro12 was used to categorize the tweets into positive, neutral, and negative categories in order to identify key factors related to each of these three categories. The results make it possible to identify the factors related to the sustainable care of the environment that are most relevant to public opinion in Social Media and organize them with regard to SDGs and the key factors about environment and public health. To this end, we relied on the methodological process proposed by Saura et al. [2] and Ekenga et al. [28].

### 4.1. Sample

In two previous studies, Bologna and Hayashi [53] and Palomino et al. [38], sentiment analysis was applied to two Twitter samples, one with 2000 tweets and another with 10,000 tweets, respectively [54,55,56]. On the other hand, Palomino et al. [38] retrieved 6333 tweets related to the hashtag #getoutside. Following these previous studies, the sample in the present study was composed of 5873 tweets that used the hashtag #WorldEnvironmentDay and were published in English on 5 June 2018 [57]. At first, the sample included 9467 tweets, but, after the process of cleaning up the database, the final sample was reduced to 5873 tweets. Retweets using #WorldEnvironmentDay were removed because it is considered duplicate content that does not enrich the database [38]. Of the users participating in the study, 47.5% were male, 52.5% were female, and no other gender was detected (as provided by the Twitter API) [58,59,60]. In addition, following Saura et al. [2], the sample of tweets was validated according to the following criteria [61]:

- Active Twitter profile (profiles without activity for three months prior to the use of #WorldEnvironmentDay were deleted)
- Twitter user profile with a profile photo and a cover
- Retweets from the same tweet about #WorldEnvironmentDay were removed (i.e., considered as duplicate content)
- Only public profiles and tweets in English using #WorldEnvironmentDay on 5 June 2018, were included
- Tweets should have been at least 80 characters long with spaces and use the tag #WorldEnvironmentDay; therefore, tweets without the “#” or a wrong label like #WorldEnvironmentDay2018 were omitted.

The tweets came from all over the world. Table 4 specifies the weight of each country from which relevant tweets were made. The three most active countries in terms of number of tweets were the United States, United Kingdom, and India. The countries with the smallest number of tweets were Malaysia, Mexico, and Belgium. 

### 4.2. Data Collection and Extraction

For the data collection and extraction process, we connected to the public Twitter API on 7 June 2018, two days after the #WorldEnvironmenDay for which we use Python software 3.7.0 (Python, Wilmington, DE, USA) in the version for MAC. This was made to ensure that all the countries of the world would have had the opportunity to publish tweets with respect to #WordEnvironmentDay for the time difference and within the capabilities of the Twitter API that only allows data to be extracted during the last 7 days of activity. The day of #WordEnvironmentDay is the day chosen by the United Nations and other global associations to highlight the care of the planet through the environment. Other purposes include carrying out social actions that demand the care of issues directly related to #WordEnvironmentDay such as global warming or pollution, and encourage new initiatives that improve the care of the planet [6].

As specified in Section 4.1, after cleaning up the database, the sample was composed of 5873 tweets. To this database, we applied the algorithm developed in Python by MonkeyLearn (San Francisco, CA, USA) [62] after connecting to its API. This was done after training the machine with data-mining processes and subdividing it into positive, negative and neutral feelings. In the datamining development process, a total of 732 samples were trained. The training of these samples of tweets was done through the MonkeyLearn application and its interface linked to the Sentiment Analysis algorithm until reaching the probability percentage of >0.650. This training of the algorithm was carried out by the authors of the research under criteria linked to the identification of ironies, sarcasms and contents related exclusively to the objective of this research. In the entire process, those contents that were not related to #WordEnvironmentDay were discarded from the sample and training. Subsequently, the different divisions of the databases according to sentiment were processed using NVivo Pro 12 Software [63] at different stages in which the tweets were categorized according to the following three nodes according to the feelings expressed by the tweets: Positive (N_1_), Neutral (N_2_), and Negative (N_3_). 

### 4.3. Sentiment Analysis and Textual Analysis

With regard to sentiment analysis, we first connected to the algorithm and trained it with data-mining processes using a sample of tweets related to the environment and sustainability until we managed to increase the significance and possibility of algorithm prediction a >0.650 (probability percentage), which is an indicator that measures the average success of a machine when using machine learning techniques [2]. The process of possibility of prediction of the algorithms that work with machine learning is an indicator that measures the effectiveness of analysis of the machine according to the feeling and can be identified in terms of the average of the database or the analysis of a single tweet [51].

The probability percentage is a measure that sets out the accuracy and recall of samples in each category. This percentage is the result of the classification success obtained by the Support Vector Machine (SVM) algorithm that works with machine learning and that was trained with data mining to perform the sentiment analysis. This percentage defines the total average success of the algorithm in the review’s classification. Similarly, it should be mentioned that we used an algorithm based on SVM typing. Supervised learning is the most popular category of machine learning algorithms. The disadvantage of this approach is the fact that, for each training example, we must provide the correct result until the algorithm gets a correct percentage of success. SVM algorithms are a non-probabilistic model that uses a representation of text examples as points in a multidimensional space. These examples are mapped so that the examples of the different categories (feelings) belong to distinct regions of this space. Then, the new texts are mapped on this same space and predicted as belonging to a category according to the region in which they are located [55,64,65].

Next, the different databases according to feelings were processed using NVivo Pro 12 at different stages in which the tweets were categorized into the following three nodes: Positive (N_1_), Neutral (N_2_), and Negative (N_3_). This process of entering the data in Nvivo is manual, although the databases are already divided in their feelings. Subsequently, the researchers proceed to make the structure of the nodes and filter the database eliminating those words as connectors, prepositions or articles and their plural forms. Then, the nodes are predefined data containers grouped according to their characteristics. It should be noted that the design and development of nodes is a norm to analyze pure data and to achieve the highest descriptive and exploratory quality possible [2]. In this sense, a relevant indicator in the analysis with NVivo Pro 12 is the weighted percentage, which represents the weight of the indicators grouped into nodes according to the times they are repeated, thus being the weight of the nods in terms of the total data that exist in the database. To calculate the weighted percentage, we used NVivo Pro 12 with the following formula (see Equation (1)):

K = ∑ki/ni = {1, …, n} n = [1, 25](1)

In the formula, K is found using a query that allows the program to search the text. The behavior of each of the words and for each tweet can be seen. Therefore, the K value was found for the Hashtag #WorldEnvironmentDay. In this way, the average K for all the tweets was calculated in order to obtain the global value [2].

## 5. Results

**Figure 1:**
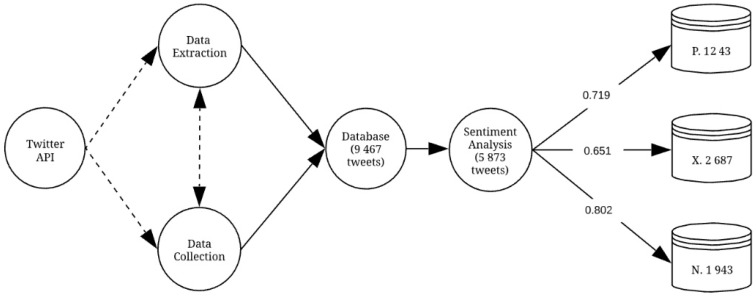
Development of the methodological process. Source: The authors.

As mentioned above and as can also be seen in Figure 1, as the first step of the methodological process, we performed data extraction and data collection from the Twitter API. After completing these processes and debugging the data, we obtained the database of n = 5873 tweets.

After applying sentiment analysis, all analyzed tweets that used #WorldEnvironmentDay were divided into Positive (P, n = 1243), Neutral (X, n = 2687), and Negative (N, n = 1943). The total mean probability coefficient of the sentiment analysis was 0.719 for P, 0.651 for X, and 0.802 for N (see Table 5). As can be seen in Table 4, the average total probability coefficient was 0.724, and it was greater than >0.650. This value needs to be considered in the analysis since it indicates the probability of success when classifying the tweets by the tool used.

**Figure 2:**
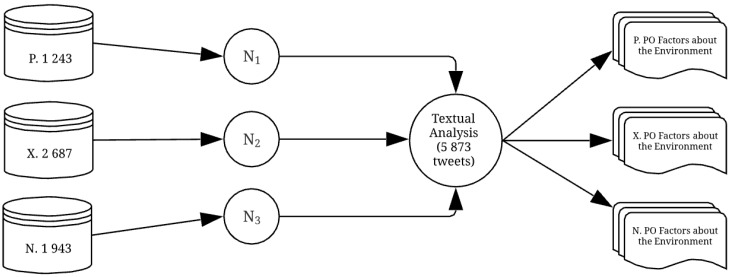
Relationship of the nodes and number of guest reviews for environment factors identification. Source: The authors.

After obtaining the three databases according to the expressed feelings, in order to identify the factors related to the environment, the data related to each feeling were added into different nodes using NVivo Pro 12. Therefore, the structure had three different nodes, N_1_ (positive), N_2_ (neutral), and N_3_ (negative). On these independent nodes, textual analysis was carried out. Textual analysis subdivided the pure data into content categories related to the environment according to the feelings expressed by the tweets. Therefore, the division of the databases into nodes yielded the following distribution of tweets in the three nodes: N_1_ = 1243 tweets, N_2_ = 2687 tweets, and N_3_ = 1943 tweets (see Figure 2).

For the structure of the nodes N_1_, N_2_, and N_3_, we also took into account the SDGs related to the environment and public health. These served as categorizers according to the feelings shown by the users in the tweets related to #WorldEnvironmentDay (for an example, see Table 6).

Likewise, the hashtags related to #WorldEnvironmentDay that refer to different actions and programs that support sustainable development, the environment, and public health were identified (see Table 7).

Specifically, one can identify topics related to climate change, the elimination of materials such as plastic and initiatives against it, programs related to oceans and fish farms, as well as other initiatives to promote the environment and sustainability in general.

The use of textual analysis yielded the following grouping of tweets into nodes according to the expressed feelings [64,65,66,67]. Results have been obtained grouped in Nodes according to feeling.

Table 8 shows the percentages corresponding to the weighted percentage indicator, as well as the roaming of the words representing the nodes and the concepts related to each of the indicators identified by textual analysis. Likewise, each of the nodes represents the sentiment of the topics related to the SDGs identified in the database of tweets corresponding to #WorldEnvironmentDay. Therefore, we can observe the category of the identified indicators, the roaming number, similar factors that give shape to the node, as well as its weighted percentage in the entire database. As a result of Sentiment Analysis and Textual Analysis processes directly from the research data, factors indicated in Table 8 are identified.

## 6. Discussion

As demonstrated in previous research, public opinion in social media and associated feelings can be measured with respect to a specific research topic in the social network Twitter. In the present study, we have demonstrated that we can identify the feelings contained in the indicators related to the key factors about environment and public health, and closely linked to the SDG, in Twitter.

As suggested by our results, there is a greater negative concern with respect to the environmental indicators included into the following SDGs: Climate Action (N_4_—Climate Change), Clean Water and Sanitation (N_4_—Water), Life on Land (N_4_—Forest), Responsible for Production (N_4_—Pollution and Biodiversity), and Sustainable Cities and Communities (Massive Industrialization). Therefore, it can be concluded that public opinion in social media is negative regarding the environment and factors such as climate change, problems with water in terms of pollution, deforestation or massive tree felling, the pollution that drives climatic changes and increases the risk of weakening of the atmosphere, biodiversity and its growing scarcity, and, finally, massive industrialization that eliminates populations and affects poor communities that face difficulties in industrialized cities. 

The negative factors that were detected may be the most useful for non-profit organizations and groups that fight for sustainable development, since public opinion in social media, through its negative evaluation, demonstrates its concern with respect to a specific issue. In our case, this topic is environmental protection materialized in the SDG. In addition, if we link the topics of the SDG with the indicators related to the environment published around #WorldEnvironmentDay, we can relate each of them to the following SDGs: Good Health and Well-being, Clean water and Sanitation, Affordable and Clean Energy, Industry Innovation and Infrastructure, Sustainable Cities and Communities, Responsible for Production, Climate Action, Life Below Water and Life on Land.

Our results also suggest that, in terms of positive indicators related to the environment, public concerns are grouped around public health, as well as clean energy and sustainability. Furthermore, in terms of neutral indicators related to the environment, there are indicators related to sustainable cities, development of sustainable resources, and promotion of a healthy lifestyle. Therefore, the factors perceived as positive are those related to the right actions taken about the environment while neutral or negative ones need to be improved. In addition, the results of the present study demonstrate that public opinion on Twitter can be measured with respect to impact issues, thus allowing to identify the key factors associated with a given topic, which can ultimately be evaluated against the agendas proposed by policy-makers. In the present study, we have shown this by analyzing the tweets with the hashtag #WorldEnvironmentDay and linking them to the SDGs and the key factors about environment and public health proposed by the United Nations.

Similarly, we have shown that Twitter is a tool that can be meaningfully used to measure sentiment regarding an emerging topic through the analysis of tweets linked to a hashtag. Therefore, social media can be regarded to be relevant venues enabling the analysis of public opinion in Social Media, which can ultimately facilitate decision-making of companies, institutions, and global communities that support sustainable development [64,65,66].

Finally, our results have demonstrated that the use of new emerging technologies, such as techniques based on machine learning and sentiment analysis algorithms developed in Python, can be useful to improve the understanding of the community of a global society, as well as to develop analysis strategies for large amounts of data.

## 7. Conclusions

In this context, new technological environments [68] and, in particular, social media, have become a reflection of the evolution of new technologies. In particular, social media have become a new channel used by people, social movements, political parties, companies, non-profit associations, or communities to express their opinion or concerns about a determined subject [2,38,69].

In the present study, focusing on the analysis of tweets with the hashtag #WorldEnvironmentDay, we have been able to identify the main factors that concern the global population with respect to the sustainable development of the planet, public health, and the environment [70,71,72].

The importance of our results is determined by the relevance of the analysis of public opinion in social media about the environment in social media. Also, considering the initiatives such as the United Nations’ development of the SDGs, the use of this technique to analyze the users’ feelings is of relevance to the scientific community and can serve to substantiate the findings obtained using other methodologies [2,38,53,72]. This fact precisely acquires relevance insofar as the contributions of this research are based on models and techniques that analyze a social network in which millions of users participate. Therefore, the use of these techniques and the results of studies of these characteristics can help to contrast studies already carried out or to identify new problems related to climate change [2,53,72]. As demonstrated by our results, the analysis of the sentiment expressed in the communications of Twitter users can help identify social, economic, environmental and cultural factors regarding their positive, neutral or negative charge [73,74,75,76]. Regarding Hypothesis 1 (H1), the results of sentiment analysis confirm that users’ feelings about the environment can be identified. This finding suggests that the Twitter platform can be used to measure the feeling of users with respect to a specific topic. Furthermore, our results also support Hypothesis 2 (H2), since textual analysis identifies the feeling of users about the environment through the study of the hashtag #WordEnvironmentDay [77]. Finally, Hypothesis 3 is supported as well, since, as has been demonstrated with the development of the methodological process, the indicators identified after textual analysis and sentiment analysis could be linked to the SDGs formulated by the United Nations.

### 7.1. Implications for the Industry

Taken together, the results of the present study can be meaningfully used by NGOs, social associations, or companies that support sustainable development programs to improve their policies and initiatives related to the environment and sustainability. Our findings can also be used by the United Nations and other institutions that see to develop or improve, where appropriate, the new SDGs in 2019. During this year, they will be reviewed again and taking into account the potential of social networks to measure public opinion on specific issues, this research can be used to improve the development of new SDGs.

In addition, policymakers can also use the results of this research to focus their future efforts on activities and actions aimed at improving the environment and combating climate change. In addition, they can confirm that social networks such as Twitter can be a good communication channel to publicize their activities and social projects on world-renowned days such as #WorldEnvironmentDay and the topics related to this event. In addition, with data offered by this study methodology, they can even improve the segmentation of their target audience in social networks to carry out concrete actions in certain countries that are concerned about the environment and that actively demonstrate this concern on Twitter.

### 7.2. Implications for Academics

The events that involve the planet in problems related to the environment have been the object of thorough research over the past decade [34,67]. This research uses relatively new methodologies, and its main innovation lies in the use of new technologies such as machine learning and datamining, and its connection with social networks such as Twitter that are known worldwide and that bring together a multitude of users around specific themes [2].

Academics can use this research as a bibliographic source not only for the environment but also for the increase of references to methodologies such as sentiment analysis applied to social networks, public opinion on social networks and the development of machine learning. In addition, within the environmental sector, the present study refers to specific research related to environmental research based on emerging technologies. In this context, we conclude one of the benefits of applying a sentiment analysis process is that there are no biases in the classification by sentiment since this is done by an algorithm and not by a researcher manually. The present study developed a methodology based on innovation that can be used in other studies with larger samples in which it is necessary to automate processes. Academics can use this study, therefore, to find references within this relatively new category of research and emerging technologies.

The indicators established in the present study convey public opinion in social media on such important issues, as global climate change, deforestation, water pollution, and unsustainable building overcrowding [72] and can be used in future research as the object of study. In addition, researchers can use the results of this study in future research to improve the methodology and focus on sectors based on technology and innovation such as Twitter and the opinions of users with respect to a specific topic in social networks.

The limitations of the present study are related to the time horizon analyzed, the limitations of the social network Twitter itself, as well as the number of users who made comments using the hashtag under study. Likewise, the limitations of the present study are also related to the level of accuracy of the algorithm trained with machine learning processes that identify the sentiments of the sample as well as the consequently establishment of the factors identified in this study.

## Figures and Tables

**Table 1:** UN Sustainable Development Goals (SDGs).

Goal	Title	Description
1	No poverty	End poverty in all its forms everywhere
2	Zero hunger	End hunger, achieve food security and improved nutrition and promote sustainable agriculture
3	Good health and well-being	Ensure healthy lives and promote well-being for all at all ages
4	Quality education	Ensure inclusive and equitable quality education and promote lifelong learning opportunities for all
5	Gender equality	Achieve gender equality and empower all women and girls
6	Clean water and sanitation	Ensure availability and sustainable management of water and sanitation for all
7	Affordable and clean energy	Ensure access to affordable, reliable, sustainable and modern energy for all
8	Decent work and economic growth	Promote sustained, inclusive and sustainable economic growth, full and productive employment and decent work for all
9	Industry, innovation, and infrastructure	Build resilient infrastructure, promote inclusive and sustainable industrialization and foster innovation
10	Reduce inequality within and among countries	Reduce inequality within and among countries
11	Sustainable cities and communities	Make cities and human settlements inclusive, safe, resilient and sustainable
12	Responsible consumption and production	Ensure sustainable consumption and production patterns
13	Climate action	Take urgent action to combat climate change and its impacts
14	Life below water	Conserve and sustainably use the oceans, seas and marine resources for sustainable development
15	Life on land	Protect, restore and promote sustainable use of terrestrial ecosystems, sustainably manage forests, combat desertification, and halt and reverse
16	Peace, justice, and strong institutions	Promote peaceful and inclusive societies for sustainable development, provide access to justice for all and build effective, accountable and inclusive
17	Partnership for the goals	Strengthen the means of implementation and revitalize the global partnership for sustainable development

Source: United Nations [6].

**Table 2:** Earthquakes and corresponding hashtags.

Hashtag (#)	Location	Date	Year	Magnitude	Dead
#FuerzaMexico	México	8 September	2017	8.2	370
#KurdistanEarthquake	Iran	12 November	2017	7.3	630
#earthquakenepal	Nepal	25 April	2015	7.8	8964
#prayingforjapan	Japan	11 March	2011	8.9	15,896
#Haiti	Haiti	12 January	2010	7.0	160,000
#Chileanearthquake	Chile	27 February	2010	8.8	525

Source: The authors based on Thapa [36] and Pena [37].

**Table 3:** Important related work.

Study	Aim	Social Network	Unit of Analysis
Saura et al. [2]	To identify the positive, neutral, and negative factors highlighted by visitors to Spanish hotels	Twitter	Posts
Cao et al. [39]	To identify behavior patterns related to time and space that explain user well-being and happiness	Twitter	Posts
Ekenga et al. [27]	To identify the issues that most concern the community about environmental risks, by analyzing the public response on the Twitter social network to the comments regarding the high amounts of water at St. Louis of Missouri	Twitter	Posts
Chisholm and O’Sullivan [42]	To illustrate that Twitter can provide information on housing as a public and social health problem, in a case study of the campaign #characterbuildings that started in New Zealand in 2014	Twitter	Hashtag
Palomino et al. [36]	To understand the impact of the natural environment on people’ well-being and health by identifying public opinion and studying the discourse related to nature-deficit disorder and other nature-heath concepts	Twitter	Hashtag
Woo et al. [43]	To examine the tweets published about a natural disaster in Korea and to identify the changes in users’ sentiment	Twitter	Posts

Source: The authors.

**Table 4:** Country percentage publishing.

Country	Percentage
United States	7.95
United Kingdom	4.64
India	4.40
Nigeria	3.97
South Africa	3.51
Kenya	3.21
Canada	3.31
Spain	1.99
France	1.32
Australia	1.32
Malaysia	1.67
Mexico	1.09
Belgium	1.02

Source: The authors.

**Table 5:** Average classification of machine learning probability percentages.

#WorldEnvironmentDay	Positive	Neutral	Negative	Total
Tweets	1243	2687	1943	5873
Average Probability	0.719	0.651	0.802	0.724

Source: The authors.

**Table 6:** Sample of the Tweet classification according to the Sentiment.

User	Tweet	Sentiment	Average Probability
@SeaShepherdSSCS	It’s World Environment Day! Did you know that much of the trash in our Oceans was improperly disposed of on land?	Negative	0.719
@VashtiHarrison	We only have one planet, hoping we’re all doing our part to take care of it	Neutral	0.671
@UNYouthEnvoy	Happy World Environment Day! Check out creative ideas to #BeatPlasticPollution from ppl all around the world	Positive	0.930
@HammalHaidar	If someone doesn’t believe in global warming, take them to Balochistan #BalochistanTurningIntoDesert	Negative	0.602

Source: The authors.

**Table 7:** Similar Hashtags and frequency of use.

Hashtags	Frequency *
#climatechange	614
#PlanetOrPlastic	460
#beatplasticpollution	314
#PlanetOrPlastic?	229
#plastic	208
#BeatPlasticPolution	98
#WorldEnvironmentDay2018	42
#WorldOceansWeek	32
#SeaLegacy	32
#GetFishFarmsOut	32
#Savetheplanet	32
#GetFishFarmsOut	23
#pollutioncrime	22
#pollution	20
#PlasticFreeLiving	14
#worldoceansday	10
#UseLessPlastic	8

* Frequency = the number of times one of the hashtags was repeated within textual analysis. Source: The authors.

**Table 8:** Results for N_1_, N_2_, and N_3_ for environment factors.

Nodes	Count	Similar Factors	Weighted Percentage
Node_1_—Positive environment factors indicators			
Public Health	207	Diseases, Health, Healthy food, Health Equity, Safe sanitation, Essential medical products	2.56
Clean Energy	195	Green energy, Solar energy, Biofuel, Biomass	2.13
Sustainability	172	Energy efficiency, renewable energies, Sustainable development	2.04
Node_2_—Neutral environment factors indicators			
Sustainable cities	286	Smartcity, Smartcities, Green urban, green architecture	2.93
Sustainable management	271	Energy management, Water management, resources sustainable management	2.79
Innovative Ideas	209	Innovative challenges, sustainable ideas	1.89
Promote well-being	151	e-Health, Healthy food, Healthy activities, Sports	1.68
Node_3_—Negative environment factors indicators			
Climate Change	378	Global warming, extreme weather, climate actions, climate change effects	4.25
Water	319	Safe water, water pollution, water conservation, water resource	4.02
Forests	251	Natural environments, Deforestation, Urban development	2.98
Pollution	230	Climate risks, preventing pollution, acid rain, atmosphere, plastic	2.75
Biodiversity	146	Plants, Trees, Animals, biological diversity	1.84
Massive Industrialization	140	Healthy homes, Malnutrition, Low carbon development	1.72

Source: The authors.
